# Pheromone and morphometric features of honey bee (*Apis mellifera*) queens imported to Canada from around the globe

**DOI:** 10.1371/journal.pone.0359657

**Published:** 2026-10-07

**Authors:** Alison McAfee, Brendan Daisley, Dylan J. L. Brettingham, Elizabeth Mallory, Christine Macpherson, Armando Alcazar Magaña, Emma Allen-Vercoe, David R. Tarpy, Leonard J. Foster

**Affiliations:** 1 Michael Smith Laboratories, Department of Biochemistry and Molecular Biology, University of British Columbia, Vancouver, British Columbia, Canada; 2 Department of Applied Ecology, North Carolina State University, Raleigh, North Carolina, United States of America; 3 Molecular and Cellular Biology, University of Guelph, Guelph, Ontario, Canada; 4 Life Sciences Institute, Department of Biochemistry and Molecular Biology, University of British Columbia, Vancouver, British Columbia, Canada; University of Leipzig Faculty of Life Sciences: Universitat Leipzig Fakultat fur Lebenswissenschaften, GERMANY

## Abstract

Canada imports approximately 300,000 honey bee queens annually to support the beekeeping industry, in addition to producing queens domestically during the short rearing season. Over 80% of imported queens come from the U.S., with additional contributions (~5% each) from Italy, Australia, and Chile. We conducted morphometric analysis and pheromone profiling of domestic and imported queens representing all four dominant exporting countries, including “vaccinated” (transgenerational immune-primed, or TGIP) and non-TGIP queens from the U.S., to compare their physical and pheromonal qualities. Imported queens differed morphometrically from domestic queens, and this difference was driven by smaller ovary size (a plastic metric exhibiting transient changes due to temporary confinement). Queen retinue pheromone (QRP) profiles also differed in domestic and imported queens, and PERMANOVA analysis showed that transit duration and ovary size were the top two explanatory factors. TGIP queens tended to have lower pheromone abundances than non-TGIP queens; a pattern that does not appear to be driven by pre-existing morphological differences. This study did not assess whether morphometric and pheromone differences affect acceptance rates or performance, nor whether immune priming offers benefits if pheromone discrepancies are enduring. By the measured laboratory metrics, domestic (Canadian) queens are comparable or better on several dimensions and investment in local production appears justified.

## Introduction

As the sole reproductive female within a honey bee colony, the health and quality of the queen has a profound influence on colony productivity [1,2], and beekeepers are motivated to maintain queens with high standards in their operations. This is particularly important at higher latitudes where colonies experience long, cold winters during which there is no opportunity for queen management. In Canada, in particular, a multiyear synthesis of national beekeeper surveys showed that “poor queens” was the most frequently reported cause of overwintering mortality overall [3]. Beyond mortality events, colonies with young queens yield more honey and produce more contiguous brood patterns than colonies with older queens [4,5], and beekeepers commonly replace queens annually to maintain this vigor. During periods of rapid population growth in the spring, beekeepers may also split colonies to rebuild populations or grow their operations, adding a new mated queen to avoid the delay and risk associated with allowing the colony to rear a queen naturally [6]. Together, these activities cumulate in a period of high demand for queens in the spring. At northern latitudes, new queens can only be produced in a short seasonal window (available typically from late May to August), which means that local queen producers are often not able to fulfill the early season queen demand [7].

To support beekeepers’ needs, Canada imports approximately 300,000 queens annually from international suppliers [8] ― predominantly the U.S., but also countries overseas ― where warmer climates or inverse seasons enable queens to be produced earlier and more reliably than in Canada. However, an unknown (but likely comparable) number of queens are also produced and sold domestically in Canada. Vulnerability to border closures due to biological risks of importation (e.g., Africanized bee genetics or novel parasites) and political factors (e.g., tariffs or war) mean that it would be prudent for the Canadian bee industry to assess the quality of domestic queen stock and invest in new methods of storing queens overwinter to meet early spring demand.

In the absence of colony-level metrics such as brood pattern and egg laying rate, queen quality is commonly assessed via morphometric measurements, stored sperm parameters, and, less commonly, by measuring pheromone production [9–20]. These parameters are generally positively correlated; for example, thorax width, wing length, and spermatheca diameter correlate with stored sperm counts [9,13], likely because a larger thorax and wings can improve queen mating flights. Colonies headed by larger, heavier queens have higher honey production [21,22] and queen size has been previously linked to increased fecundity [23], possibly due to their increased likelihood of mating success [1]. However, relationships among morphological characteristics and fecundity are not always consistent across datasets [18,20], and additional quality indicators should be considered.

Queen retinue pheromone (QRP) ― the complete suite of which is composed of 9-oxo-2(E)-decenoic acid (9-ODA), 9-R- and 9-S-hydroxydec-2(E)-enoic acid (9-HDA), 4-hydroxy-3-methoxyphenylethanol (HVA), methyl p-hydroxybenzoate (HOB), methyl (Z)-octadec-9-enoate (MO), (Z9,Z12,Z15)-octadeca-9,12,15-trienoic acid (LEA), hexadecan-1-ol (PA), and (E)-3-(4-hydroxy-3-methoxyphenyl)-prop-2-en-1-ol (CA) ― is the best studied honey bee queen pheromone blend, though notably, other cuticular hydrocarbons appear also to be biologically active with at least some functional overlap [24]. QRP appears to be linked to queen quality to some degree; however, similar to the physical parameters, relationships are not always clear. High-quality queens sometimes, but not always, produce more 9-HDA, HOB, and HVA than low-quality queens [19], and mating triggers changes in pheromone profiles, but not all studies agree on which components are altered and in which direction [25–27]. QRP is clearly important and necessary for colony cohesion, but the factors regulating its production are still not well understood.

It is well-known that confined (caged) queens may experience extended laying disruptions during shipping and storage ahead of sale, leading to ovary size (area) reduction [28,29]. This small-ovary phenotype is reversible and is neither indicative of future performance [30,31] nor queen acceptance [32]; rather, it is likely an indicator primarily of the queen’s instantaneous egg-laying status. Ovary area and mass are functions of both the number of ovarioles (a developmentally determined feature that is fixed throughout a queen’s life) as well as whether those ovarioles are actively producing oocytes. The relative contributions of these two features cannot be distinguished from area or mass measurements alone; nonetheless, there is precedent for using these metrics in reproductive assessments (e.g., [18,29,30,33–38]). Domestic queens have previously been found to have heavier ovaries than international queens [18], which was likely an effect of differential caging and transport durations. However, all else being equal, ovary area and weight are still useful reproductive metrics because they are related to other important features such as the level of virus infection [30] and pheromone production [32], particularly MO, which contributes to inhibiting queen cell rearing [38]. Any assessment of queen quality from multiple shipping origins should include a range of metrics in addition to ovary parameters to ensure that potential differences are not solely an effect of caging duration.

Queens that have been subject to transgenerational immune priming (TGIP) — colloquially referred to as being “vaccinated” — to prevent American foulbrood (AFB) infection are a recent product offered commercially. However, little is known about how TGIP (which is achieved indirectly by feeding attendant workers a formula containing bacterial fragments [39]) may or may not influence other aspects of the queen’s physiology. Such an effect is conceivable, given that the process of vaccination is expected to stimulate the queen’s immune system, and queens can exhibit a trade-off between reproduction and immunity in other contexts [30,31]. While AFB resistance is beneficial, and vaccination may yield secondary benefits of colony-level resistance to other pathogens [40], there is also a need to evaluate potential physiological trade-offs.

Our aim in this study was to survey domestic and imported queens from a range of globally distributed origins. In an exploratory analysis, we measured morphometrics and QRP profiles of 95 queens from four Canadian provinces (British Columbia, Alberta, Ontario, and Quebec, making up the “domestic” import category) and four international sources outside of Canada (United States, Chile, Australia, and Italy, making up the “international” import category), including TGIP and non-TGIP queens from the U.S. We hypothesized that ovary sizes would differ strongly among import categories, given the expected dependency on caging and shipping durations, but that other metrics would be similar. Due to previously identified relationships among queen immune stimulation, ovary mass, and production of QRP components [30,37,38], we further hypothesized that TGIP queens may differ from non-TGIP queens in one or more quality parameters. Finally, we capitalized on this rich dataset of physical and pheromonal quality metrics to test whether QRP profiles are better correlated with reproductive metrics or body size metrics. These data do not address queen acceptance or performance within colonies; rather, we conduct physical and pheromonal laboratory assessments to broadly assess the quality metrics, and relationships therein, of queens that are commercially available to Canadian beekeepers.

## Methods

### Queen sampling and morphometric measurements

A total of 95 queen bees from 17 independent suppliers were procured during the spring of 2024 from large-scale commercial breeders across Canada (British Columbia, Alberta, Ontario, and Quebec) and internationally (United States, Chile, Australia, and Italy; Fig 1). See S1 File for complete sample metadata. The sample sizes were n = 5 each for BC, AB, and QC queens (all mixed ancestry), n = 20 Buckfast queens from ON, n = 15 mixed ancestry queens from ON, n = 5 Italian queens from ON, n = 5 Carniolan queens from Italy, n = 5 Buckfast queens from Italy, n = 15 non-TGIP queens from the U.S., and n = 10 TGIP queens from the U.S. Among the U.S. non-TGIP queens, ancestries included n = 10 Carniolan and n = 5 Saskatraz hybrid queens. Among the U.S. TGIP queens, ancestries included n = 5 Carniolan and n = 5 Italian queens. Except in the case of queens from ON, supplier and shipment batch are largely confounded with country origin and ancestry. The intended value of these data is therefore not to draw conclusions around specific countries or ancestries, but overall patterns of more broadly grouped domestic and international queens, as well as grouped TGIP and non-TGIP queens from the U.S. For these reasons, and due to the limited sample size per country (n = 5 in some cases), country-wise differences were not assessed.

**Fig 1:**
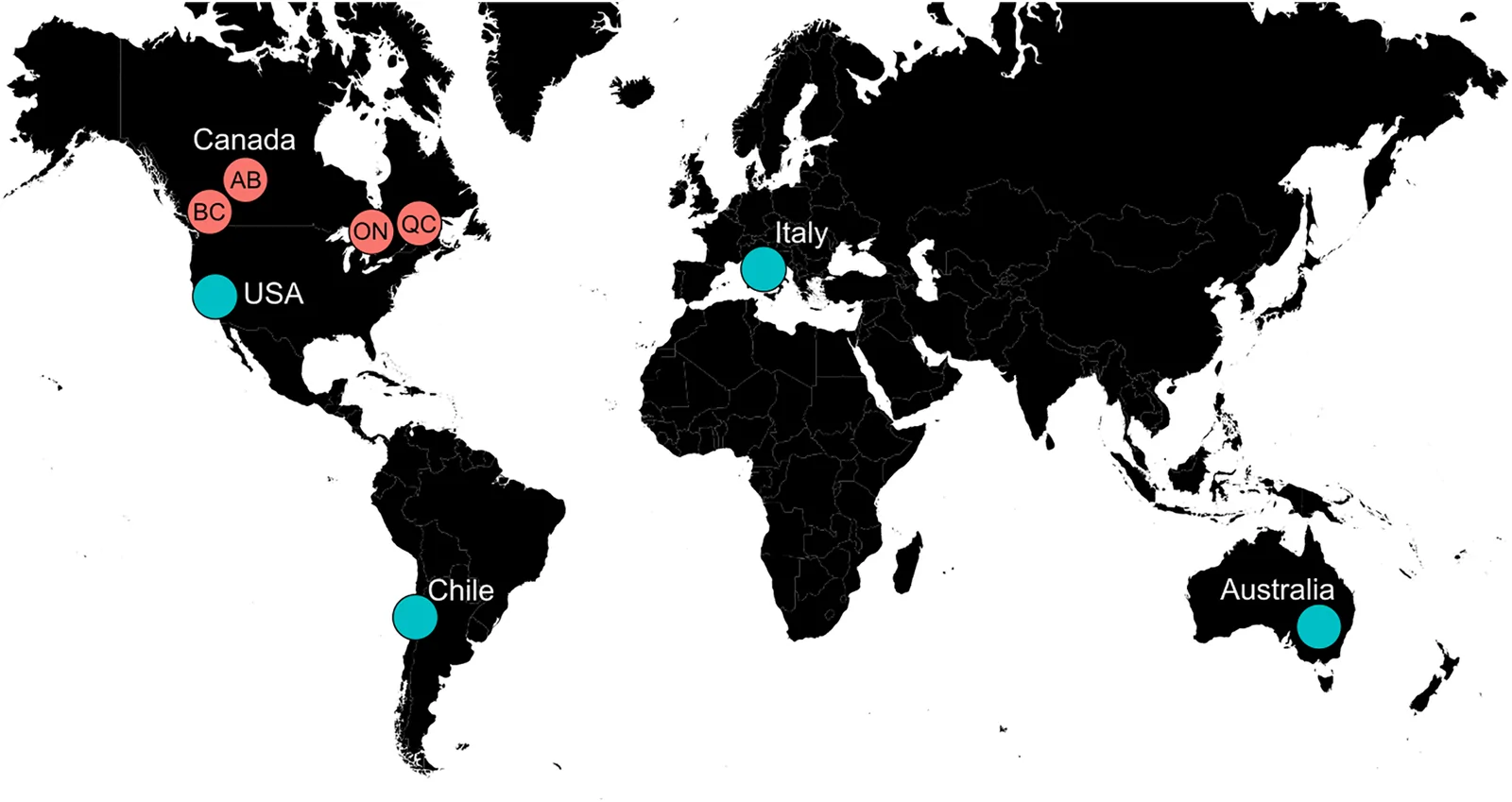
Sources of Canadian (domestic) and international (imported) queens obtained for this study. A) Domestic queens were obtained from four provinces: British Columbia (BC), Alberta (AB), Ontario (ON) and Quebec (QC). International queens were obtained from four countries (US, Chile, Italy, and Australia). Map vector file obtained from www.mapsvg.com (license: CC0 1.0 Universal).

Live queens were shipped via express postal services to the University of Guelph with a transit time of 1–5 days, depending on the point of origin. Exact age of the queens is unknown, but given standard saleable queen rearing and practices, they are expected to be young (~1 month old). Upon arrival, all queens were cryogenically killed at –20 °C for a minimum of 30 minutes prior to downstream analyses. We evaluated 9 morphological traits known to reflect queen quality, including total body weight, head width and length, thorax width and length, basitarsus width and length, ovary size (area), and spermatheca size (area), based on established metrics from previous studies [13,20,41].

Each queen’s total body weight was measured using an analytical balance with ±0.1 mg precision (Sartorius Canada). Dissections were then performed to isolate the ovaries, spermatheca, and right hindleg. Tissues were positioned anatomically on 5% agarose gels, and high-resolution dorsal-side digital images were captured before and after each dissection, with a physical ruler included for scale. Image analysis was conducted using ImageJ software (v1.54). Briefly, the “Set Scale” function was used to calibrate pixel measurements to physical distances using a 25 mm segment of the in-frame ruler, measuring the distance from the left-most edge of the markings. This established the spatial scale for the active image such that all subsequent measurements were reported in calibrated units (±0.01 mm). Linear dimensions (e.g., width and length of the head and basitarsus) were then obtained using the line tool. The areas of the spermatheca and each ovary were determined by outlining the structures with the ellipse or polygon tools, respectively, and applying the “measure area” function.

Thorax lengths and widths were measured at the time of dissection using digital calipers. The caliper jaws were aligned across the maximum distance between the lateral margins of the pronotum (i.e., the widest point at the forewing insertion region), with the caliper held perpendicular to the longitudinal body axis to ensure consistent orientation. For thorax length, the caliper jaws were aligned along the dorsal midline extending from the most anterior point of the prothorax (near head-thorax junction) to the posterior edge of the propodeum (i.e., the anatomical length of the thoracic region visible in dorsal view). This approach captures the anteroposterior dimension of the thorax as a straight-line measurement. All measurements were recorded to the nearest 0.01 mm in technical triplicate to ensure precision and consistency between samples. This method aligns with standard practice in insect morphometrics, where linear body dimensions are quantified directly on physical specimens with digital calipers at defined anatomical landmarks [9,42–44]. Head, thorax, and basitarsus “size” estimates were calculated by multiplying length and width measurements. These calculations are not intended to represent the total area, given that the body segments are not rectangular; rather, this approach enabled us to account for variation in both length and width measurements while reducing the total number of metrics tested in subsequent analyses.

### Pheromone extraction and identification

Queen heads were shipped on dry ice to the University of British Columbia for pheromone analysis following procedures as previously described [32]. Briefly, we followed a two-phase extraction protocol [45] to extract bulk lipids (upper, nonpolar fraction) and metabolites (lower, polar fraction) from each queen head sample. The samples, including five blanks processed in parallel (no bee tissue added), were homogenized with ceramic beads in 400 µl of 75% methanol, 0.01% butylated hydroxytoluene extraction solution, then 1 mL of methyl-tert-butyl ether (MTBE) was added. The methanol extraction solution also contained 4 internal standards (1 lipid and 3 metabolites, respectively): CUDA (12- [[(cyclohexylamino)carbonyl]amino]-dodecanoic acid), caffeine-^13^C_3_, ferulic acid-d3, and methionine-d3. The samples were incubated with agitation for 1.5 h at room temperature. Upon addition of dH_2_O, the samples were vortexed and centrifuged to complete phase separation. 250 µl of the upper lipid fraction was then reserved and stored at −70°C until liquid chromatography-tandem mass spectrometry (LC-MS/MS) analysis. The lower metabolite fraction was not analyzed in this study. Please see the previously published methods [32], updated LC parameters [38], and accompanying step-by-step protocol for sample preparation (https://benchling.com/s/prt-WrKhN14IeaOuFgIoEyYH?m=slm-ifWOAUee9IS5DNLSNfSC) for further procedural details.

Directly ahead of analysis, lipid samples were evaporated to dryness and reconstituted in 70% acetonitrile, 30% isopropanol injection solvent, then analyzed by a Vanquish UHPLC system (Thermo) equipped with an ACQUITY UPLC CSH C18 analytical column (130 Å, 1.7 µm, 2.1 mm × 100 mm, Waters) coupled to an Impact II QTOF mass spectrometer (Bruker). Samples were injected in randomized order. See our previous publication for specific UHPLC gradient details and mass spectrometer settings [38]. The improvements to data acquisition parameters described therein enabled the seven pheromone targets to be identified entirely in the lipid fraction, rather than across both lipid and metabolite fractions. External standards of each pheromone component (10 ppm) were analyzed separately to confirm retention times and spectra of target compounds. Peak areas were normalized by a factor of total ion abundance within the Progenesis QI software (version 3.0.7600.27622) with the METLIN plugin (version 1.0.7642.33805, NonLinear Dynamics) to enable relative quantification. The internal lipid standard (CUDA) was used to monitor the stability of instrument response across runs and was not used directly as a normalization denominator. The “all ions” Progenesis QI normalization approach is more statistically robust, removing only the offset that all ions share and not the differences specific to individual compounds [46]. The resulting normalized values represent relative compound abundances that enable comparisons of the same compound across conditions, but not comparisons of one compound to another due to the well-known limitations of mass spectrometry (most notably, chemistry-dependent variation in ionization efficiency).

### Statistical analysis

All statistical analyses were conducted in R (version 4.3.0) using R Studio (version 2023.09.1 + 494) [47]. Differences in ancestry were often not distinguishable from differences in country origin and queen supplier; therefore, only supplier was included as a factor in our analyses. Actual sample sizes may differ from the number of queens assessed, depending on the analysis, because 9 ON queens and 1 BC queen had missing spermatheca measurements due to poorly contrasting images. An AB queen and two ON queens have missing ovary area data for the same reason. TGIP queens were only included in analyses explicitly addressing effects of vaccination and were excluded from other tests.

Pearson correlation matrices among morphometric and, separately, pheromone variables were assessed for statistical significance using the Holm method of multiple hypothesis testing correction. Next, principal component analyses (PCAs) were conducted on scaled and centered variables using the prcomp function. Scaled pheromone and morphometric profiles were then separately modelled using PERMANOVA analyses using the adonis2 function (specifying 9999 permutations, assessing the marginal effects of terms, and Euclidean distance) from the vegan R package [48]. The effect of import category (2 levels: domestic and international) effects was first evaluated in the absence of additional covariates. Supplier effects could not be reasonably accounted for as a fixed factor in the PERMANOVA analyses because it consumed too many degrees of freedom, and it could not be included as a blocking factor because supplier is nested within import category, preventing the necessary permutations. Supplier is, however, accounted for in subsequent analyses (see below). To investigate relationships between pheromone profiles and morphometrics, we used a reductive modelling approach where import category, transit duration, and all morphometrics were initially included in the PERMANOVA model and non-significant predictors were sequentially removed. The final model included transit duration, ovary size, and basitarsus size as predictors. No significant dispersion associated with explanatory factors were detected. In cases where continuous variables were used as predictors, they were converted into categorical bins solely to test for dispersion.

Individual morphometrics and pheromone compounds were compared among import categories using the aligned rank transform ANOVA test [49] (a non-parametric approach which was necessary due to unequal variances and poorly fitting linear models for some response variables). Import category was the sole fixed effect and supplier was included as a random intercept term. Transit duration (days) was compared between import categories using the Wilcoxon rank sum test. The relationship between ovary size and transit duration was evaluated using a linear model with transit duration as a predictor and supplier as a fixed effect. A preliminary analysis of ovary size that instead used supplier as a random effect, which would have been preferable, showed that residual distributions were not adequate, whereas no significant residual deviations were detected with supplier modelled as a fixed effect. Relationships between specific pheromones and morphometrics were assessed using linear models, initially using the same model structure arrived by the reductive PERMANOVA analysis strategy (that is, with transit duration, ovary size, and basitarsus size included as fixed effects) in addition to supplier as a fixed effect to improve residual distributions. Basitarsus size was subsequently removed because it was not significantly linked to abundance of any specific pheromone, and residual distributions were further improved in its absence. Residuals resulting from all parametric tests, here and throughout, were assessed using diagnostic tests within the DHARMa package [50] to confirm appropriateness of fit.

A subset of ON queens, where queens from 8 suppliers were received in a single shipment batch, were used to test for supplier variation in morphometric and pheromone profiles by PERMANOVA analysis. In this case, queens were all in the same import category and had the same transit duration, so only supplier was included as an explanatory factor. We did identify significant beta dispersion (p = 0.046) when modelling the ON pheromone data, but inspection of a diagnostic PCA showed that one supplier did completely separate from the rest, indicating that a false positive effect of supplier was unlikely.

Morphometric and pheromone profiles of TGIP and non-TGIP queens were assessed using a PERMANOVA with TGIP group as a fixed effect. These queens were supplied as a single shipment batch with two contributing suppliers, but TGIP queens are represented by only one supplier; therefore, supplier could not be included in this model. Individual group (TGIP vs. non-TGIP) comparisons for each pheromone compound were conducted using Welch’s t test (to account for unequal variances) instead of the aligned rank transform ANOVA as previously employed because no random effects were needed. P values were assessed for significance with respect to α = 0.0071 (the Bonferroni method for multiple hypothesis testing correction). Appropriateness of residual distributions was confirmed in every instance. Morphometrics were not individually compared because no significant profile differences were detected by PERMANOVA analysis.

## Results

### Morphometric differences in domestic and imported queens

Overall, queen weight averaged at 204.6 ± 26.1 mg (s.d.), ovary size at 17.1 ± 7.1 mm^2^ (i.e., 34.2 mm^2^ total), spermatheca size at 1.15 ± 0.17 mm^2^, head width at 3.72 ± 0.15 mm, head length at 3.59 ± 0.22 mm, thorax width at 4.33 ± 0.32 mm, thorax length at 4.51 ± 0.31 mm, basitarsus width at 1.20 ± 0.07 mm, and basitarsus length at 2.46 ± 0.11 mm. Raw and aggregated data for morphometrics and pheromones are available in S1 File. Correlations among morphometrics are shown in S1A Fig, where only queen weight and ovary area were significantly correlated after multiple hypothesis testing correction (Holm method). Principal component analysis (PCA) of the morphometric array revealed modest separation of domestic and international suppliers (Fig 2A). The morphometric principal components did not clearly separate into primarily reproductive vs. body size metrics; PC1 (explaining 28.9% of the variation) is most influenced by head size and PC2 (explaining 25.3% of the variation) is most influenced by ovary size, while the remaining morphometrics (thorax size, basitarsus size, and spermatheca size) contribute substantially to both PCs.

**Fig 2:**
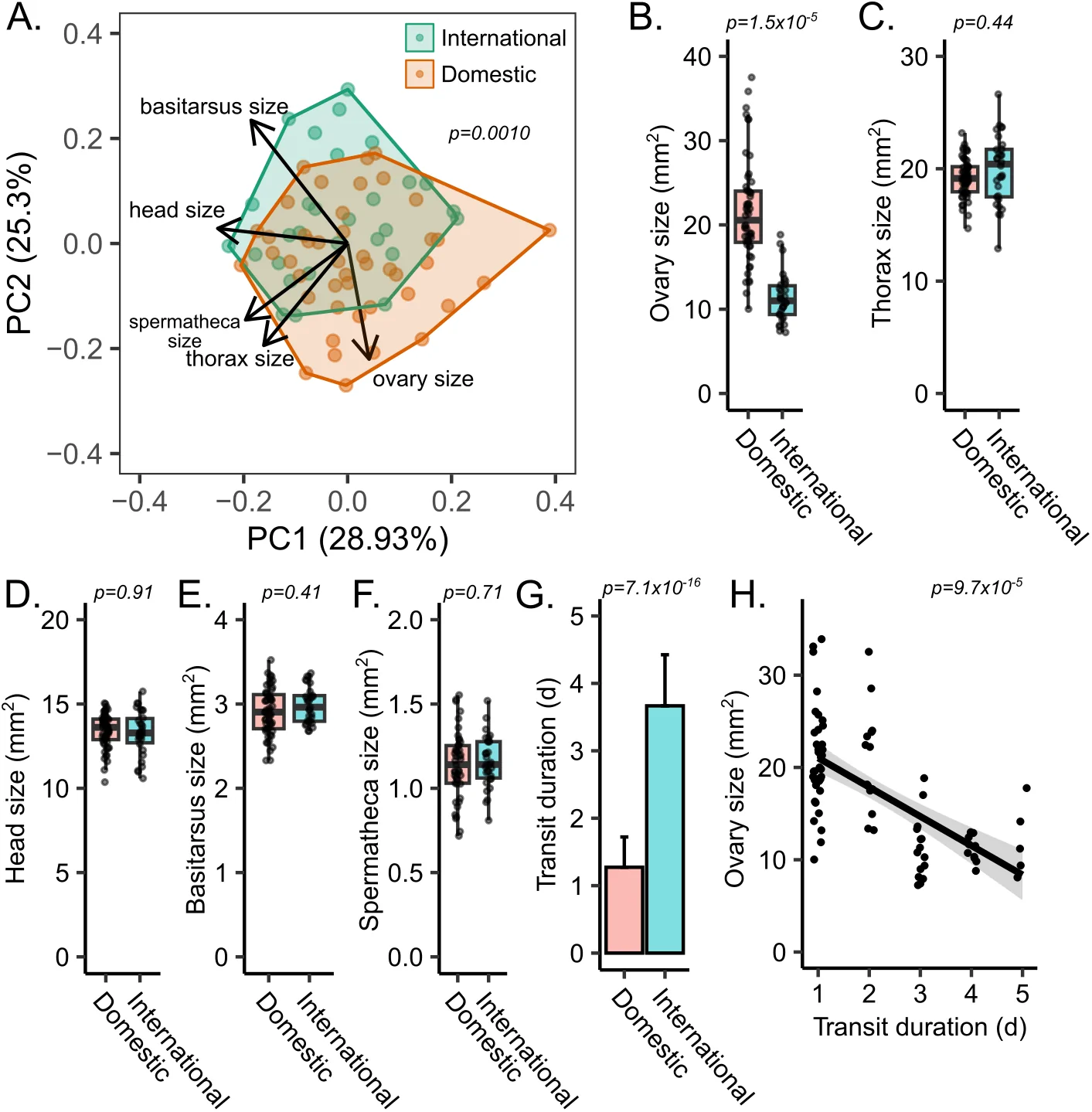
Morphometric differences in domestic and international queens. A) Principal component analysis reveals modest but significant morphometric separation of domestic vs. international queens (PERMANOVA, F_1,70_ = 9.5, p = 0.001). B-G) Boxes represent the interquartile range, bars indicate the median, and whiskers span 1.5 times the interquartile range. Ovary size was the only metric significantly differing between domestic and international queens (aligned rank transform ANOVA, F_1,13.3_ = 44, p = 1.5x10^-5^). G) International queens experienced a 2.4 d longer transit duration than domestic queens (Wilcoxon rank sum test, W = 0, p = 7.1x10^-16^). Error bars indicate standard deviations. H) Ovary size is negatively linked to transit duration (linear model, estimate = −4.5, p = 9.7x10^-5^). Grey shading indicates the standard error.

PERMANOVA analysis comparing domestic (n = 43) and international (n = 29) queens confirmed that, while separation is modest in the PC space, the morphometric differences are statistically significant (N = 72, F_1,70_ = 9.5, p = 0.001). Inspecting each morphometric individually (Fig 2B-F), the difference is most strongly influenced by the ovaries, which are approximately twice as large in domestic queens compared to international queens, consistent with previous findings [18]. Ovary size was the only individual morphometric significantly differing between domestic and imported queens (aligned rank transform ANOVA, N = 82, F_1,13.3_ = 44, p = 1.5x10^-5^; α = 0.010 via Bonferroni correction).

We hypothesized that the ovaries of imported queens were smaller than domestic queens due to different caging and travel times, during which the ovaries temporarily shrink [28,29]. Indeed, all domestic queens experienced transit durations of 1–2 d, whereas international queens experienced transit durations of 3–5 d, resulting in 2.4 d longer travel time, on average (Wilcoxon rank sum test, N = 85, W = 0, p = 7.1x10^-16^; Fig 2G). Transit duration is thus completely confounded with domestic vs. international import categories. Regressing ovary size against transit duration revealed a significant ovary area loss of approximately 4.5 mm^2^ per day (linear model, N = 82, estimate = −4.5, p = 9.7x10^-5^; Fig 2H) while accounting for supplier effects, supporting the hypothesis that ovary area changes as a consequence of travel/caging time and may not indicate an inherent reproductive advantage of domestic queens.

### Pheromone differences in domestic and imported queens

We analysed seven QRP compounds (9-ODA, 9-HDA, HVA, HOB, MO, CA, and LEA) in all queen samples and identified no statistically significant correlations among them after multiple hypothesis testing correction (Holm method; S1B Fig). QRP profiles differed between domestic (n = 55) and imported (n = 30) queens (PERMANOVA, N = 85, F_1,83_ = 10.5, p = 0.0010; Fig 3A; example chromatograms in S2 Fig; relative quantitation data in S1 File). Subsequent inspection of each compound shows that only LEA significantly differed between import categories after multiple hypothesis testing correction (aligned rank transform ANOVA, N = 85, F_1,13.2_ = 23, p = 0.00035, α = 0.0071 via Bonferroni correction), which was elevated in domestic queens. LEA is obtained from pollen [51], and we speculate that it may have been higher in domestic queens due to the queen’s attendants having more recent pollen access.

**Fig 3:**
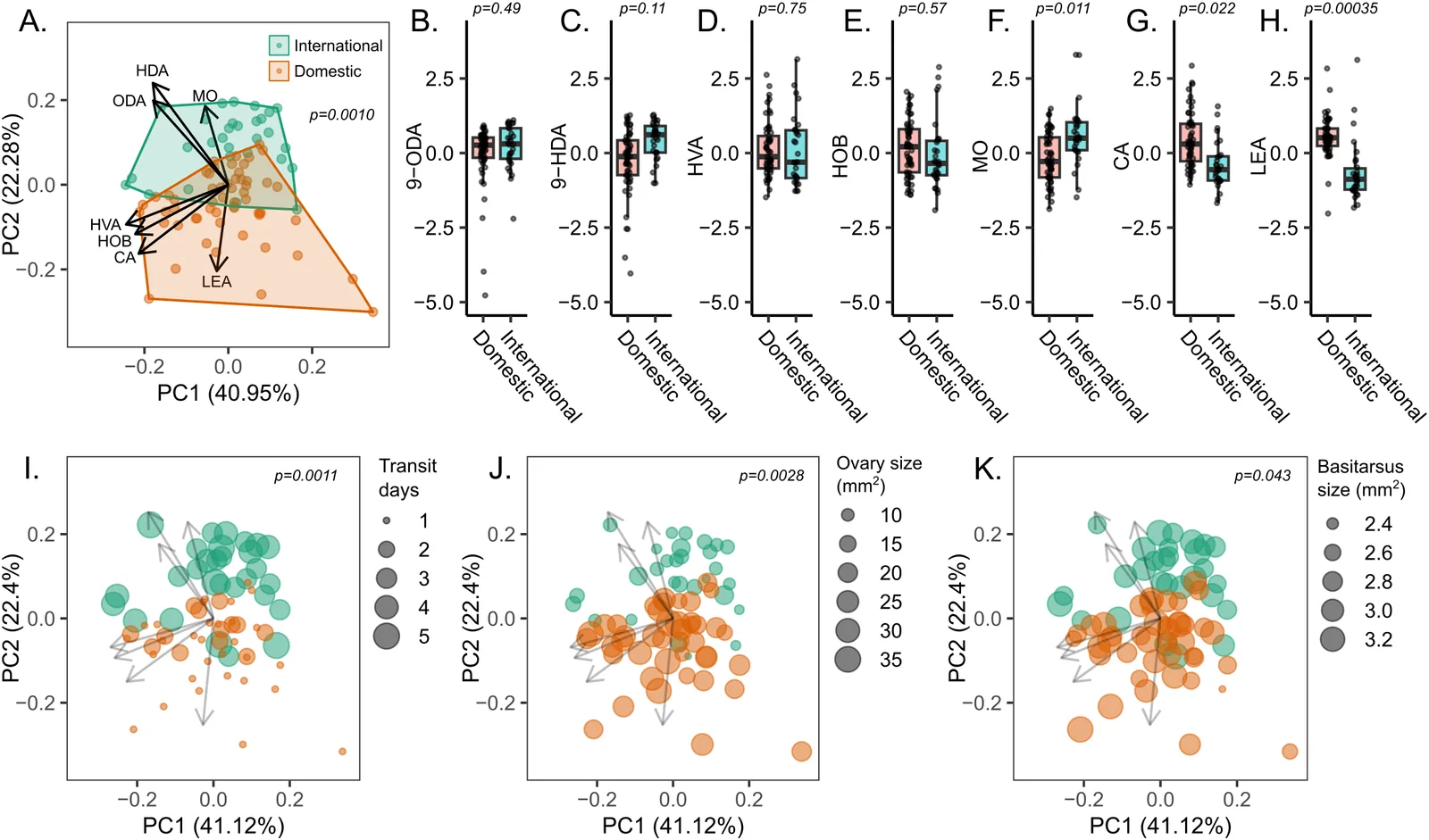
Pheromone differences in domestic and international queens. A) PCA of QRP compound abundances shows divergence by queen import category. The difference is statistically significant (PERMANOVA, F_1,83_ = 10.5, p = 0.0010). B-H) Boxes represent the interquartile range, bars indicate the median, and whiskers span 1.5 times the interquartile range. Scaled pheromone abundances are shown. LEA was the only individual compound significantly differing between import categories after multiple hypothesis testing correction (aligned rank transform ANOVA, F_1,13.2_ = 23, p = 0.00035, α = 0.0071). I) Reductive modeling of pheromone profiles shows that transit duration (PERMANOVA, F_1,68_ = 5.2, p = 0.0011) is a better explanatory factor than import category, which was no longer a significant predictor when both terms were included in the model. J) Ovary size was the dominant morphometric predictor of pheromone profiles (F_1,68_ = 4.4, p = 0.0028). K) Basitarsus size was also a significant predictor but the effect magnitude was smaller (F_1,68_ = 2.5, p = 0.043).

We hypothesized that, if QRP components are indicators of reproductive quality as has been previously suggested [11,19,52], QRP profiles should be better explained by explicit reproductive characteristics than body size characteristics (which can also be tied to reproduction, but indirectly). Because morphometric PCs did not neatly delineate into reproductive and non-reproductive components to use as predictors, we instead used a reductive modelling approach where import category, transit duration, plus all morphometric variables were initially included in a PERMANOVA model and predictors with the highest p value were sequentially removed. We found that ovary size was the most significant morphometric predictor (N = 72, F_1,68_ = 4.4, p = 0.0028), partially supporting our hypothesis. This effect was significant despite the inclusion of transit duration, which was also a significant predictor (F_1,68_ = 5.2, p = 0.0011), indicating that the relationship between QRP profiles and ovary size does not appear to be solely a consequence of caging time. Basitarsus size was also significantly linked to QRP profiles (F_1,68_ = 2.5, p = 0.043), but was not the dominant morphometric predictor. Examining each pheromone compound individually (while accounting for supplier as a fixed effect) showed that 9-ODA was, surprisingly, positively linked to transit duration (linear model, estimate = 1.5, p = 2.5x10^-9^, α = 0.0071), in addition to 9-HDA (estimate = 1.6, p = 9.1x10^-11^). No individual pheromones were significantly linked to ovary size after multiple hypothesis testing correction, but HVA and HOB were marginally non-significant in this regard (estimate = 0.056, p = 0.010 and estimate 0.049, p = 0.013, respectively). Similar results were obtained by running the models again without transit duration; therefore, this lack of relationship is not due to collinearity of ovary size and transit duration.

### Supplier effects on morphometric and pheromone profiles

Because queens from some countries were obtained in one shipment batch from one supplier, we could not determine how much variation originated from supplier effects, location effects, and batch effects throughout these analyses. To gain insight into the level of supplier-induced variation, we analyzed a subset of queens from ON, Canada, which were represented by eight suppliers where all queens were provided in a single shipment batch and therefore had the same transit duration. Among these queens, PERMANOVA analysis revealed a significant supplier effect on QRP profiles (N = 40, F_7,29_ = 4.6, p = 0.0010) as well as morphometrics (N = 30, F_7,22_ = 1.6, p = 0.043). Results involving groups represented by only one supplier, such as the TGIP queens assessed below, should therefore be interpreted cautiously.

### TGIP queens have depleted pheromone profiles but similar morphometrics to non-TGIP queens

“Vaccinated” (transgenerational immune-primed, or TGIP) queens are an emerging tool to help prevent AFB disease in colonies [39,40]. Since transgenerational immune priming involves exposing the queen to bacterial fragments, we hypothesized that this may stimulate the immune system, leading to smaller ovaries and a reduction in the QRP component MO (methyl oleate; a phenomenon we have previously observed upon stimulating queens’ immune systems using virus infections [30]). PCA of TGIP (n = 10) and non-TGIP (n = 15) queens shows that there is substantial overlap in morphometrics, with just two TGIP queens falling outside the non-TGIP frame (Fig 4A), agreeing with previous findings [53]. Accordingly, we detected no morphometric differences (PERMANOVA, N = 25, F_1,23_ = 1.1, p = 0.34). We observed modest separation of QRP profiles (Fig 4B), and PERMANOVA analysis shows that this slight divergence of QRP profiles is statistically significant (N = 25, F_1,23_ = 3.8, p = 0.0082). QRP components were generally depleted in TGIP queens (Fig 4C-I), and while no individual differences survived multiple hypothesis testing at α = 0.0071 (Bonferroni), the most significant and largest magnitude difference was in methyl oleate (Welch’s t test, t = 2.7, estimated df = 18, p = 0.014), partially supporting our initial hypothesis. Since there were no differences in morphometric profiles, the general QRP depletion appears not to be linked to a reduction in ovary size or other pre-existing differences in static morphometrics.

**Fig 4:**
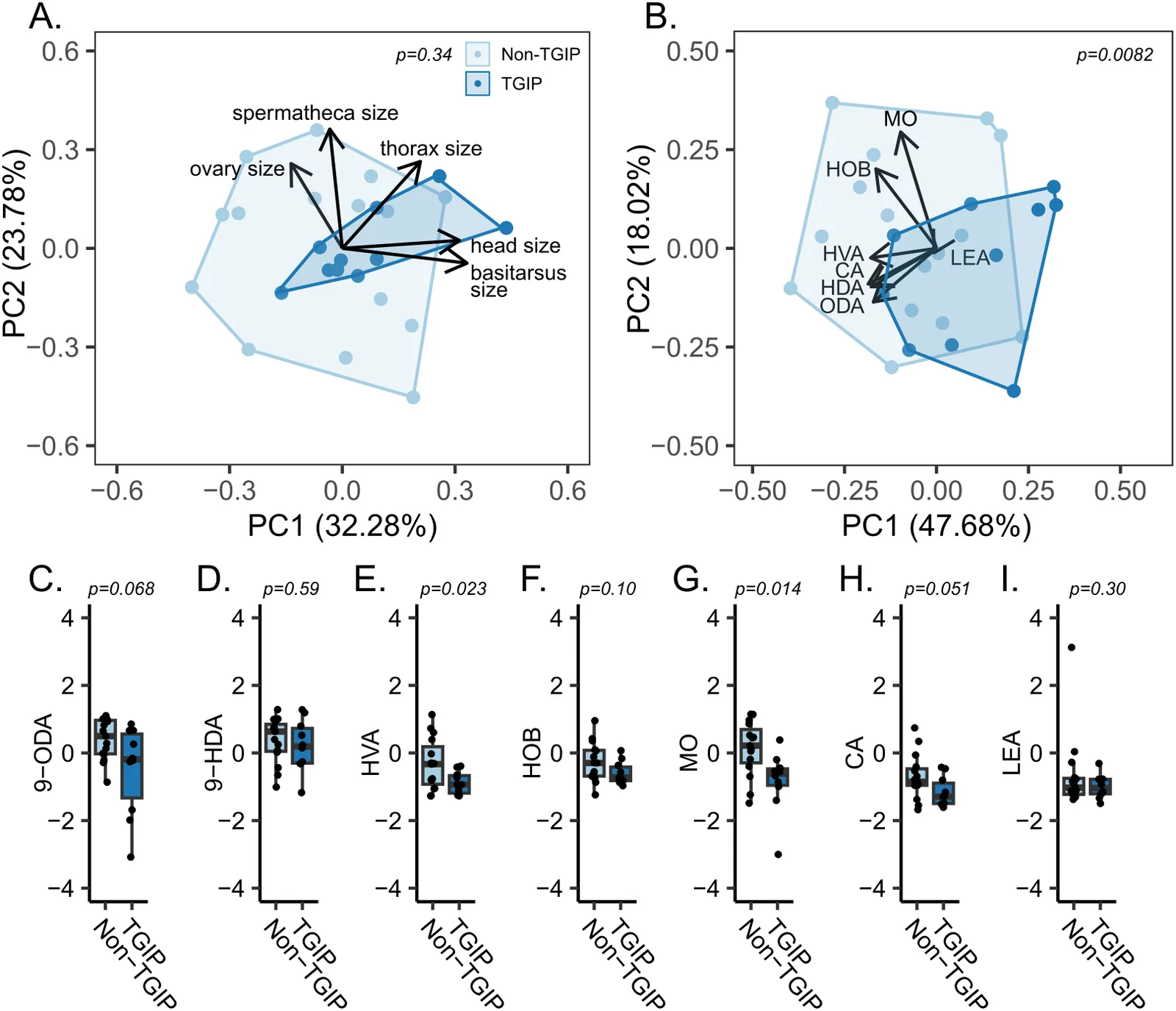
TGIP queens have different pheromone profiles. A) Principal component analysis (PCA) of morphometrics shows that TGIP and non-TGIP queens overlap substantially, and PERMANOVA analysis confirms no differences (PERMANOVA, F_1,23_ = 1.1, p = 0.34). B) PCA of pheromone profiles show modest but significant separation between vaccination groups (F_1,23_ = 3.8, p = 0.0082). C-I) TGIP queens tend to have lower pheromone abundances but no differences were significant (Welch’s t tests) after multiple hypothesis testing correction (α = 0.0071, Bonferroni). Boxes represent the bounds between the 2nd and 3rd interquartile range, midlines represent the median, and whiskers are extended by 1.5 times the interquartile range.

## Discussion

Here we present a global dataset of queen quality metrics. Our analysis uncovered four key findings: 1) Physically, domestic (Canadian) and internationally-sourced queens differed only in that Canadian queens had larger ovary sizes, which is an expected outcome due to longer shipping durations of queens from distant origins; 2) QRP profiles also varied between Canadian and international queens, with LEA being found at higher abundance in Canadian queens; 3) Ovary size was the dominant morphometric predictor of pheromone profiles; and 4) TGIP queens tended to display depleted QRP quantities compared to non-TGIP queens. These results carry important implications for the growth of the domestic queen trade and knowledge of QRP production dynamics. We analyzed queens from five countries in this dataset, but it is important to explicitly note that the primary value of this study is not a comparison of queen quality from different countries, since one batch of as few as five queens from one supplier does not accurately represent queens from an entire country; rather, the more valuable comparisons are the broader multi-batch assessments of domestic and international queens as well as the overall relationships between QRP and morphometric features.

Our morphometric correlations differ from some previous datasets, but this is easily explained once the sampling context is taken into account. Tarpy et al. [54] and Delaney et al. [9] both found a significant positive correlation between queen body mass and thorax width, whereas we found a positive but non-significant correlation (S1A Fig). Notably, in these prior studies, the context of queen sampling (where queens were either reared under controlled settings or were collectively banked ahead of sampling) was substantially more uniform than in ours, where queens were shipped from multiple international origins and sampled on the day of receipt. This means that our sampled queens experienced different caging durations, which significantly affected their ovary size, as highlighted in Figs 2G and 2H. Ovary size and queen weight were significantly positively correlated in our data, as one might expect, and this is likely why we did not identify a significant link between queen weight and thorax width here, since shipping differences obscure the relationship.

Since these queens were purchased from commercial producers with whom we do not have direct contact, we were unable to account for supplier differences in queen age and handling (e.g., banking duration, production methods, etc.) prior to shipping. We have explored some of these factors in an independent set of locally-produced queens previously [32], but since the exact parameters are unknown here, this may contribute to variation among queen suppliers. Genetic stock (e.g., Italian, Carniolan, Saskatraz, mixed ancestry, etc.) may also contribute to supplier variation but in many cases is indistinguishable from supplier in this dataset. In this analysis, queen supplier thus represents an aggregate factor that is likely influenced by genetic, producer, age, and environmental effects. Such differences may still be considered inherent properties contributing to the characteristics of the pool of queens that are commercially available to Canadian beekeepers.

Overall, Canadian and international queens are of comparable quality (Fig 2–3) and Canadian queens have competitive physiological standards. Coupled with prior data showing that local breeding stocks exhibit higher overwintering survival rates [18] and honey production [55] than international stocks, this supports the notion that expanding Canadian queen production is worthwhile and should, biologically, continue to meet or exceed beekeepers’ needs. The ongoing challenge of Canadian queen supply, however, is the timing and reliability of production [7]. Substantial progress has been made in overwintering queens en masse in queen banks [56–58], and future work should build on this success and diversify queen storage methods.

The variation of pheromone abundances we observed in this dataset challenges the notion of using a standardized dose of pheromone compounds blended into what is commonly referred to as a “queen equivalent” or “Q_eq_”, as it has previously been defined [59] in experiments investigating pheromonal effects. Our diverse samples illustrate that we have a poor grasp of what an average queen equivalent actually is, even among queens tested in similar reproductive contexts (i.e., all experiencing restricted laying and comparable worker attendance). In our data, relative abundances of QRP components span a 9-fold to 233-fold range, depending on the compound, with 9-ODA being the most stable and HOB being the most variable (S1 File). By contrast, relative abundances of our internal standard for the lipid fraction (CUDA), which represents technical variation, spanned just a 3-fold range. This level of technical inconsistency is not ideal, but the relative difference between CUDA and QRP component variation indicates that the variation in QRP data is largely due to biological differences. While standardization is important to enable comparisons between studies, in some cases it may be better to define what a queen equivalent is empirically, as there are clearly factors influencing QRP production that we do not yet understand, in addition to known seasonal [60] and age-related differences [61,62].

One such previously unknown factor is the influence of virus infection on QRP production, particularly the MO component [38]. In controlled infection experiments with black queen cell virus and deformed wing virus B, the infection (or perhaps the associated immune stimulation) caused queens’ ovaries to shrink and MO to decline [38]. This is relevant to the present discussion because, comparing TGIP and non-TGIP queens (the former of which are exposed to an immune stimulating product in the form of inactivated bacterial fragments [39]), we found that relative QRP abundances generally declined with TGIP, with MO showing the largest difference (Fig 4). Morphometrics, including ovary size, in the two groups were similar, which is consistent with our previous observation of a diminished effect on ovaries when queens were injected with inactivated virus particles compared to live inocula [30]. Importantly, honey bee queens possess other pheromones that are at least in part functionally redundant with some QRP components [24]; these additional compounds were not investigated here but could conceivably mask TGIP-induced depletions. Future work should aim to investigate this “vaccine effect” more rigorously. In particular, we do not have ancestry- and supplier-matched samples in both TGIP and non-TGIP groups, which are necessary to eliminate genetic and effects and other variables not inherently linked to TGIP treatment. If our observed patterns are validated, potential mechanisms of TGIP-induced QRP suppression and the duration of this effect should be investigated. Existing evidence suggests that TGIP queens are, overall, beneficial [39,40], but further exploration of this topic is nonetheless warranted.

Our initial hypothesis that QRP profiles would be more strongly linked to reproductive morphometrics than body size morphometrics was partially supported by the finding that ovary size was a significant positive predictor of pheromone abundance (Fig 3J); a relationship that we confirmed is not an artifact of differential transit duration. This is consistent with previous findings that total ovary mass predicted queen mandibular pheromone amounts [11], and positive relationships between ovary mass and HVA [32] as well as MO [38], though not all studies agree [52]. In the present work, relationships between specific pheromones and ovary size did not survive multiple hypothesis testing correction, but the trends were positive and the effect on overall profiles was significant. Ovary areas are influenced by both the number of total ovarioles, which is developmentally determined and fixed throughout a queen’s lifetime, as well as the degree to which those ovarioles are actively producing eggs (i.e., ovariole activation). Only ovariole activation is expected to be influenced by transit duration (longer transit = less ovariole activation). The fact that ovary area was still a significant positive predictor of pheromone profiles despite the inclusion of transit duration as a covariate implies that the number of ovarioles may have been influential. We did not obtain this information in the present study, but these results suggest that being able to differentiate between ovariole number and ovariole activation will be valuable. Surprisingly, we found that transit duration actually increased 9-ODA and 9-HDA amounts through a yet unknown mechanism. Whether these pheromonal changes affect introduction success, positively or negatively, is yet undetermined.

## Conclusion

Our data suggest that QRP and morphometric parameters of domestic and imported queens are distinct and are linked to transit duration, but there is a large degree of unexplained variation in the dataset. Given that these queens were sampled after experiencing similar physical and social contexts (other than transit duration), an exploration of additional correlates, including but not limited to genetic makeup, pathogen infections, and microbiome differences, is needed. The significant QRP depletion in TGIP queens compared to non-TGIP queens suggests that there may be yet unappreciated impacts of this treatment on queen physiology, but this pattern should be confirmed among additional TGIP queen suppliers. Whether there is a lasting impact of this depletion on queen performance seems unlikely but is yet to be determined. Regardless, the TGIP queen system could provide an opportunistic scenario to test pheromonal and temporal impacts of immune stimulation using a controlled, non-infectious treatment via a socially-regulated route of exposure. Our analysis of relationships among QRP, reproductive morphometrics, and body size morphometrics suggests that the association between QRP profiles and ovary size is deeply rooted in queen biology, beyond tightly controlled experiments. More detailed measurements of sperm parameters, queen introduction success, performance within colonies, and additional non-QRP queen pheromones are necessary to fully test relationships between pheromones and reproduction, but our data provide compelling insights derived from a large sample of globally diverse queens.

## Supporting information

S1 Fig Pearson correlation matrices. Asterisks indicate significant p values (Holm method for multiple hypothesis testing correction). A) Morphometric measurements. B) Pheromone measurements. ODA = E-9-oxodec-2-enoic acid; HDA = 9(R/S)-hydroxydec-2(E)-enoic acid, MO = methyl oleate, LEA = linolenic acid, CA = coniferyl alcohol, HOB = methyl p-hydroxybenzoate, HVA = 4-hydroxy-3-methoxyphenylethanol.
(DOCX)

S2 Fig Example chromatograms of target compounds. A) Base peak chromatogram of the full LC-MS/MS run. The orange line depicts a representative quality control sample (a pool of all 95 extracts) while the black line represents a pooled external standard sample containing the target compounds. B) 9-ODA, i.e., 9-oxo-2(E)-decenoic acid (sodium adduct). C) 9-HDA, i.e., 9-hydroxy-2-decenoic acid, and 10-HDA, i.e., 10-hydroxy-2-decenoic acid (sodium adducts). D) HVA, i.e., 4-hydroxy-3-methoxyphenylethanol, also known as homovanillyl alcohol (sodium adduct). E) CA, i.e., (E)-3-(4-hydroxy-3-methoxyphenyl)-prop-2-en-1-ol, also known as coniferyl alcohol (sodium adduct). F) MO, i.e., methyl (Z)-octadec-9-enoate, also known as methyl oleate (sodium adduct). G) HOB, i.e., Methyl p-hydroxybenzoate (sodium adduct). H) LEA, i.e., (Z9,Z12,Z15)-octadeca-9,12,15-trienoic acid, also known as α-linolenic acid (ammonium adduct).
(DOCX)

S1 File Morphometric and pheromone data. Excel file containing all data underlying the figures contained within this manuscript are provided, along with group-wise summary statistics and analytical information (retention time, mass-to-charge) of the pheromone targets.
(XLSX)

## References

[1] Amiri E, Strand MK, Rueppell O, Tarpy DR. Queen Quality and the Impact of Honey Bee Diseases on Queen Health: Potential for Interactions between Two Major Threats to Colony Health. Insects. 2017;8(2):48. doi: 10.3390/insects8020048 28481294 PMC5492062

[2] Amiri E, Abou-Shaara H, McAfee A. The effect of major abiotic stressors on honey bee (*Apis mellifera* L.) queens and potential impact on their progeny. Apidologie. 2024;56(1). doi: 10.1007/s13592-024-01133-0

[3] Daisley B, Macpherson CV, Brettingham DJL, Moore AE, Thompson GJ, Allen-Vercoe E. Impacts of antibiotic use, air pollution and climate on managed honeybees in Canada. Nat Sustain. 2025;8(9):1087–99. doi: 10.1038/s41893-025-01603-y

[4] Hora ZA, Yadeta GL, Hundie TA. Young honeybee (*Apis mellifera bandasii*) queens perform better than old ones. Am J Life Sci. 2020;7(6):128–32.

[5] Winston M. Swarming, afterswarming, and reproductive rate of unmanaged honeybee colonies (*Apis mellifera*). Insectes Sociaux. 1980;27(4):391–8.

[6] vanEngelsdorp D, Tarpy DR, Lengerich EJ, Pettis JS. Idiopathic brood disease syndrome and queen events as precursors of colony mortality in migratory beekeeping operations in the eastern United States. Prev Vet Med. 2013;108(2–3):225–33. doi: 10.1016/j.prevetmed.2012.08.004 22939774

[7] Bixby M, Hoover SE, McCallum R, Ibrahim A, Ovinge L, Olmstead S, et al. Honey Bee Queen Production: Canadian Costing Case Study and Profitability Analysis. J Econ Entomol. 2020;113(4):1618–27. doi: 10.1093/jee/toaa102 32484511 PMC7313926

[8] AAFC. Statistical overview of the Canadian honey and bee industry 2024. Horticulture Section, Crops and Horticulture Division, Government of Canada. 2025. Available from: https://agriculturecanadaca/en/sector/horticulture/reports

[9] Delaney DA, Keller JJ, Caren JR, Tarpy DR. The physical, insemination, and reproductive quality of honey bee queens (*Apis mellifera* L.). Apidologie. 2011;42(1):1–13. doi: 10.1051/apido/2010027

[10] Lee KV, Goblirsch M, McDermott E, Tarpy DR, Spivak M. Is the Brood Pattern within a Honey Bee Colony a Reliable Indicator of Queen Quality?. Insects. 2019;10(1):12. doi: 10.3390/insects10010012 30626029 PMC6359415

[11] Margarita O, Osnat M, Abraham H. Choosing the best: honeybee workers can assess reproductive quality of the queen through pheromonal signalling in simultaneous choice assays. Apidologie. 2020;51(3):291–306. doi: 10.1007/s13592-019-00712-w

[12] De Souza DA, Huang MH, Tarpy DR. Experimental improvement of honey bee (*Apis mellifera*) queen quality through nutritional and hormonal supplementation. Apidologie. 2019;50(1):14–27. doi: 10.1007/s13592-018-0614-y

[13] Hatjina F, Bieńkowska M, Charistos L, Chlebo R, Costa C, Dražić MM, et al. A review of methods used in some European countries for assessing the quality of honey bee queens through their physical characters and the performance of their colonies. J Apic Res. 2014;53(3):337–63. doi: 10.3896/ibra.1.53.3.02

[14] Jackson JT, Tarpy DR, Fahrbach SE. Histological estimates of ovariole number in honey bee queens, *Apis mellifera*, reveal lack of correlation with other queen quality measures. J Insect Sci. 2011;11:82. doi: 10.1673/031.011.8201 21870968 PMC3398436

[15] Mattila HR, Reeve HK, Smith ML. Promiscuous honey bee queens increase colony productivity by suppressing worker selfishness. Curr Biol. 2012;22(21):2027–31. doi: 10.1016/j.cub.2012.08.021 23022065

[16] Milone JP, Tarpy DR. Effects of developmental exposure to pesticides in wax and pollen on honey bee (*Apis mellifera*) queen reproductive phenotypes. Sci Rep. 2021;11(1):1020. doi: 10.1038/s41598-020-80446-3 33441911 PMC7806648

[17] Williams GR, Troxler A, Retschnig G, Roth K, Yañez O, Shutler D, et al. Neonicotinoid pesticides severely affect honey bee queens. Sci Rep. 2015;5:14621. doi: 10.1038/srep14621 26459072 PMC4602226

[18] Holmes LA, Ovinge LP, Kearns JD, Ibrahim A, Wolf Veiga P, Guarna MM, et al. Queen quality, performance, and winter survival of imported and domestic honey bee queen stocks. Sci Rep. 2023;13(1):17273. doi: 10.1038/s41598-023-44298-x 37828046 PMC10570385

[19] Rangel J, Böröczky K, Schal C, Tarpy DR. Honey Bee (*Apis mellifera*) Queen Reproductive Potential Affects Queen Mandibular Gland Pheromone Composition and Worker Retinue Response. PLoS One. 2016;11(6):e0156027. doi: 10.1371/journal.pone.0156027 27281328 PMC4900659

[20] Facchini E, De Iorio MG, Turri F, Pizzi F, Laurino D, Porporato M, et al. Investigating Genetic and Phenotypic Variability of Queen Bees: Morphological and Reproductive Traits. Animals (Basel). 2021;11(11):3054. doi: 10.3390/ani11113054 34827786 PMC8614377

[21] Nelson DL, Gary NE. Honey Productivity of Honeybee Colonies in Relation to Body Weight, Attractiveness and Fecundity of the Queen. J Apic Res. 1983;22(4):209–13. doi: 10.1080/00218839.1983.11100589

[22] Lu H, Zhang X, Wei B, Wang G, You X, Qu X, et al. Body Size and Body Weight in *Apis cerana*: Associations with Geographic, Climatic, and Productive Traits for Bee Breeding. Life (Basel). 2026;16(6):980. doi: 10.3390/life16060980 42355506 PMC13301450

[23] Yu L, He X, Shi X, Yan W, Wu X. Honey bee maternal effects improve worker performance and reproductive ability in offspring. Front Cell Dev Biol. 2023;11:1156923. doi: 10.3389/fcell.2023.1156923 37181751 PMC10167042

[24] Princen SA, Oliveira RC, Ernst UR, Millar JG, van Zweden JS, Wenseleers T. Honeybees possess a structurally diverse and functionally redundant set of queen pheromones. Proc Biol Sci. 2019;286(1905):20190517. doi: 10.1098/rspb.2019.0517 31213188 PMC6599994

[25] Plettner E, Otis GW, Wimalaratne PDC, Winston ML, Slessor KN, Pankiw T, et al. Species- and Caste-Determined Mandibular Gland Signals in Honeybees (*Apis*). J Chem Ecol. 1997;23(2):363–77. doi: 10.1023/b:joec.0000006365.20996.a2

[26] Slessor KN, Kaminski LA, King GG, Winston ML. Semiochemicals of the honeybee queen mandibular glands. J Chem Ecol. 1990;16(3):851–60. doi: 10.1007/BF01016495 24263600

[27] Richard F-J, Tarpy DR, Grozinger CM. Effects of insemination quantity on honey bee queen physiology. PLoS One. 2007;2(10):e980. doi: 10.1371/journal.pone.0000980 17912357 PMC1989138

[28] Laidlaw HH, Page RE. Queen rearing and bee breeding. United States: Wicwas Press; 1997.

[29] Aamidor SE, Cardoso-Júnior CAM, Harianto J, Nowell CJ, Cole L, Oldroyd BP, et al. Reproductive plasticity and oogenesis in the queen honey bee (Apis mellifera). J Insect Physiol. 2022;136:104347. doi: 10.1016/j.jinsphys.2021.104347 34902433

[30] Chapman A, McAfee A, Tarpy DR, Fine J, Rempel Z, Peters K, et al. Common viral infections inhibit egg laying in honey bee queens and are linked to premature supersedure. Sci Rep. 2024;14(1):17285. doi: 10.1038/s41598-024-66286-5 39068210 PMC11283550

[31] McAfee A, Chapman A, Pettis JS, Foster LJ, Tarpy DR. Trade-offs between sperm viability and immune protein expression in honey bee queens (*Apis mellifera*). Commun Biol. 2021;4(1):48. doi: 10.1038/s42003-020-01586-w 33420325 PMC7794525

[32] McAfee A, Magaña AA, Foster LJ, Hoover SE. Differences in honeybee queen pheromones revealed by LC-MS/MS: Reassessing the honest signal hypothesis. iScience. 2024;27(10):110906. doi: 10.1016/j.isci.2024.110906 39391732 PMC11465126

[33] Camiletti AL, Percival-Smith A, Croft JR, Thompson GJ. A novel screen for genes associated with pheromone-induced sterility. Sci Rep. 2016;6:36041. doi: 10.1038/srep36041 27786267 PMC5081541

[34] Galang KC, Croft JR, Thompson GJ, Percival-Smith A. Analysis of the *Drosophila melanogaster* anti-ovarian response to honey bee queen mandibular pheromone. Insect Mol Biol. 2019;28(1):99–111. doi: 10.1111/imb.12531 30159981

[35] Smith AR, Seid MA, Jiménez LC, Wcislo WT. Socially induced brain development in a facultatively eusocial sweat bee *Megalopta genalis* (Halictidae). Proc Biol Sci. 2010;277(1691):2157–63. doi: 10.1098/rspb.2010.0269 20335213 PMC2880158

[36] Garcia Bulle Bueno F, Gloag R, Latty T, Ronai I. Irreversible sterility of workers and high-volume egg production by queens in the stingless bee *Tetragonula carbonaria*. J Exp Biol. 2020;223(Pt 18):jeb230599. doi: 10.1242/jeb.230599 32737215

[37] Chapman A, Amiri E, Han B, McDermott E, Rueppell O, Tarpy DR, et al. Fertility costs of cryptic viral infections in a model social insect. Sci Rep. 2022;12(1):15857. doi: 10.1038/s41598-022-20330-4 36151143 PMC9508145

[38] McAfee A, Chapman A, Alcazar Magaña A, Marshall KE, Hoover SE, Tarpy DR, et al. Elevated virus infection of honey bee queens reduces methyl oleate production and destabilizes colony-level social structure. Proc Natl Acad Sci U S A. 2025;122(42):e2518975122. doi: 10.1073/pnas.2518975122 41086214 PMC12557728

[39] Dickel F, Bos NMP, Hughes H, Martín-Hernández R, Higes M, Kleiser A, et al. The oral vaccination with *Paenibacillus larvae* bacterin can decrease susceptibility to American Foulbrood infection in honey bees-A safety and efficacy study. Front Vet Sci. 2022;9:946237. doi: 10.3389/fvets.2022.946237 36325099 PMC9618583

[40] Weinberg I, Floyd A, Reid N, Swift N. Reduction of deformed wing virus-B levels in colonies of the honey bee *Apis mellifera* after queen vaccination with inactivated *Paenibacillus larvae*. bioRxiv. 2024;2024:01.601551. doi: 10.1101/2024.07.01.601551

[41] Slater GP, Yocum GD, Bowsher JH. Diet quantity influences caste determination in honeybees (*Apis mellifera*). Proc Biol Sci. 2020;287(1927):20200614. doi: 10.1098/rspb.2020.0614 32453984 PMC7287363

[42] Skandalis DA, Tattersall GJ, Prager S, Richards MH. Body Size and Shape of the Large Carpenter Bee, *Xylocopa virginica* (L.) (Hymenoptera: Apidae). J Kansas Entomol Soc. 2009;82(1):30–42. doi: 10.2317/jkes711.05.1

[43] Tarpy DR, Keller JJ, Caren JR, Delaney DA. Assessing the mating “health” of commercial honey bee queens. J Econ Entomol. 2012;105(1):20–5. doi: 10.1603/ec11276 22420250

[44] Pemberton RW, Wheeler GS, Madeira PT. Bee (Hymenoptera: Apidae) Pollination of *Vanilla planifolia* in Florida and Their Potential in Commercial Production. Florida Entomologist. 2023;106(4). doi: 10.1653/024.106.0404

[45] Chen S, Hoene M, Li J, Li Y, Zhao X, Häring H-U, et al. Simultaneous extraction of metabolome and lipidome with methyl tert-butyl ether from a single small tissue sample for ultra-high performance liquid chromatography/mass spectrometry. J Chromatogr A. 2013;1298:9–16. doi: 10.1016/j.chroma.2013.05.019 23743007

[46] Black B, da Silva LBR, Hu G, Qu X, Smith DFQ, Magaña AA, et al. Glutathione-mediated redox regulation in *Cryptococcus neoformans* impacts virulence. Nat Microbiol. 2024;9(8):2084–98. doi: 10.1038/s41564-024-01721-x 38956248 PMC11930340

[47] R Core Team. R: A language and environment for statistical computing. Vienna, Austria: R Foundation for Statistical Computing; 2023.

[48] Oksanen J, Simpson G, Blanchet F, Kindt R, Legendre P, Minchin P, et al. Vegan: Community ecology package. R package version 26-4. 2022. Available from: https://CRANR-projectorg/package=vegan

[49] Wobbrock JO, Findlater L, Gergle D, Higgins JJ. The aligned rank transform for nonparametric factorial analyses using only anova procedures. In: Proceedings of the SIGCHI Conference on Human Factors in Computing Systems. 2011. p. 143–6.

[50] Hartig F. DHARMa: Residual diagnostics for hierarchical (multi-level/mixed) regression models. R package version 046. 2022. Available from: https://CRANR-projectorg/package=DHARMa

[51] Corby-Harris V, Bennett MM, Deeter ME, Snyder L, Meador C, Welchert AC, et al. Correction to: Fatty acid homeostasis in honey bees (*Apis mellifera*) fed commercial diet supplements. Apidologie. 2022;53(1). doi: 10.1007/s13592-022-00917-6

[52] Kocher SD, Richard F-J, Tarpy DR, Grozinger CM. Queen reproductive state modulates pheromone production and queen-worker interactions in honeybees. Behav Ecol. 2009;20(5):1007–14. doi: 10.1093/beheco/arp090 22476212 PMC2744121

[53] Gomaa S, El-Gohary E-G, Barakat E, Salama M. Morphometric analysis of some external and internal body characteristics of honey bee *Apis mellifera* queens treated with *Paenibacillus larvae* larvae. Afr J Biol Sci. 2018;14(1):1–10. doi: 10.21608/ajbs.2018.65271

[54] Tarpy DR, Keller JJ, Caren JR, Delaney DA. Experimentally induced variation in the physical reproductive potential and mating success in honey bee queens. Insect Soc. 2011;58(4):569–74. doi: 10.1007/s00040-011-0180-z

[55] Guarna MM, Hoover SE, Huxter E, Higo H, Moon K-M, Domanski D, et al. Peptide biomarkers used for the selective breeding of a complex polygenic trait in honey bees. Sci Rep. 2017;7(1):8381. doi: 10.1038/s41598-017-08464-2 28827652 PMC5566959

[56] Levesque M, Rousseau A, Giovenazzo P. Impacts of indoor mass storage of two densities of honey bee queens (*Apis mellifera*) during winter on queen survival, reproductive quality and colony performance. J Apic Res. 2023;62(2):274–86. doi: 10.1080/00218839.2022.2126613

[57] Rousseau A, Giovenazzo P. Successful Indoor Mass Storage of Honeybee Queens (*Apis mellifera*) during Winter. Agriculture. 2021;11(5):402. doi: 10.3390/agriculture11050402

[58] Lamothe Boudreau M, Giovenazzo P. Physiological qualities of honey bee queens (*Apis mellifera*) overwintered in banks. J Apic Res. 2025;65(1):145–57. doi: 10.1080/00218839.2025.2558395

[59] Keeling CI, Slessor KN, Higo HA, Winston ML. New components of the honey bee (*Apis mellifera* L.) queen retinue pheromone. Proc Natl Acad Sci U S A. 2003;100(8):4486–91. doi: 10.1073/pnas.0836984100 12676987 PMC153582

[60] Carroll MJ, Brown NJ, Ruetz Z, Ricigliano VA, Anderson KE. Honey bee retinue workers respond similarly to queens despite seasonal differences in Queen Mandibular Pheromone (QMP) signaling. PLoS One. 2023;18(9):e0291710. doi: 10.1371/journal.pone.0291710 37768918 PMC10538780

[61] Rhodes JW, Lacey MJ, Harden S. Changes with age in queen honey bee (*Apis mellifera*) head chemical constituents (Hymenoptera: Apidae). Sociobiology. 2007;50(1):11–22.

[62] Bortolotti L, Costa C. Chapter 5: Chemical communication in the honey bee society. In: Mucignat-Caretta C, editor. Neurobiology of Chemical Communication. Boca Raton, U.S.A.: Taylor & Francis; 2014. 24830041

