# Supplementary material for: Pheromone and morphometric features of honey bee (Apis mellifera) queens imported to Canada from around the globe
Source: PLoS One. 2026 Oct 7;21(10):e0359657. doi: 10.1371/journal.pone.0359657 (PMC13645040; doi:10.1371/journal.pone.0359657)
Supplement: Supplementary file 1 [file pone.0359657.s001.docx]

**S1 Fig.** **Pearson correlation matrices.** Asterisks indicate significant p values (Holm method for multiple hypothesis testing correction). A) Morphometric measurements. B) Pheromone measurements. ODA = E-9-oxodec-2-enoic acid; HDA = 9(R/S)-hydroxydec-2(E)-enoic acid, MO = methyl oleate, LEA = linolenic acid, CA = coniferyl alcohol, HOB = methyl p-hydroxybenzoate, HVA = 4-hydroxy-3-methoxyphenylethanol.
