# Supplementary material for: Pheromone and morphometric features of honey bee (Apis mellifera) queens imported to Canada from around the globe
Source: PLoS One. 2026 Oct 7;21(10):e0359657. doi: 10.1371/journal.pone.0359657 (PMC13645040; doi:10.1371/journal.pone.0359657)
Supplement: Supplementary file 2 [file pone.0359657.s002.docx]

**S2 Fig. Example chromatograms of target compounds.** A) Base peak chromatogram of the full LC-MS/MS run. The orange line depicts a representative quality control sample (a pool of all 95 extracts) while the black line represents a pooled external standard sample containing the target compounds. B) 9-ODA, i.e. 9-oxo-2(E)-decenoic acid (sodium adduct). C) 9-HDA, i.e. 9-hydroxy-2-decenoic acid, and 10-HDA, i.e. 10-hydroxy-2-decenoic acid (sodium adducts). D) HVA, i.e. 4-hydroxy-3-methoxyphenylethanol, also known as homovanillyl alcohol (sodium adduct). E) CA, i.e. (E)-3-(4-hydroxy-3-methoxyphenyl)-prop-2-en-1-ol, also known as coniferyl alcohol (sodium adduct). F) MO, i.e. methyl (Z)-octadec-9-enoate, also known as methyl oleate (sodium adduct). G) HOB, i.e. Methyl p-hydroxybenzoate (sodium adduct). H) LEA, i.e. (Z9,Z12,Z15)-octadeca-9,12,15-trienoic acid, also known as α-linolenic acid (ammonium adduct).
